# Japanese trends in breastfeeding rate in baby-friendly hospitals between 2007 and 2010: a retrospective hospital-based surveillance study

**DOI:** 10.1186/1471-2393-13-207

**Published:** 2013-11-15

**Authors:** Takashi Yoda, Kenzo Takahashi, Yoshitada Yamauchi

**Affiliations:** 1 Department of Pediatrics, Nerima Hikarigaoka Hospital, 2-11-1 Hikarigaoka, Nerima-ku, Tokyo 179-0072, Japan; 2 Department of Epidemiology and Public Health, School of Medicine, Yokohama City University, 3-9 Fukuura, Kanazawa-ku, Yokohama-shi, Kanagawa 236-0004, Japan; 3 Department of Nursing, School of Health Sciences, Kibi International University, 8 Igacho, Takahashi-shi, Okayama 716-8508, Japan; 4 The Japan Breastfeeding Association, 3-9-4 Arai, Nakano-ku, Tokyo 165-0026, Japan

**Keywords:** Breastfeeding rate, Baby-friendly hospitals, Length of stay, Japan

## Abstract

**Background:**

The goal of Japan’s national “Healthy and Happy Family 21” campaign is to increase the nationwide breastfeeding rate for babies in the first month of life, which is currently below 50%, to a level of 60%. In this article, we summarize the breastfeeding rate for all of Japan’s baby-friendly hospitals (BFHs) and extract their strengths in conjunction with the structural and legislative support that they have in place and finally draw up a policy for dispersing BFH activities to non-BFH delivery facilities, which could be useful for increasing the breastfeeding rate.

**Methods:**

This study included all of the 61 BFHs that are registered in Japan. These hospitals account for approximately 2% of nearly 3,000 Japanese delivery facilities. The surveillance data, which were collected anonymously by the Japan Breastfeeding Association in 2007–2010, were summarized. The numbers of babies who were breastfed after delivery, at discharge from BFHs and at one month of age, were collated. The length of hospital/clinic stay was also collected.

**Results:**

The collection rate was 100% in each year (2007, 2008, 2009 and 2010). The breastfeeding rates during hospital stay, at discharge, and one month were >70%, ~90%, and >75%, respectively. The median length of stay was 5 days (minimum/maximum: 5/8) for primipara.

**Conclusions:**

The breastfeeding rate at BFHs at one month of age was more than 75%. This surpassed the current national average (<50%). The median length of hospital/clinic stay was 5 days. In this 5-day period, BFH activities can play an important role in increasing the breastfeeding rate. Since hospitalization for the reported national median length of stay of 6 days, is legally guaranteed, the disbursement of BFH activities to non-BFH delivery facilities, with special support to mothers who delivered by cesarean delivery, would be a useful strategy for achieving a 60% breastfeeding rate at one month of age.

## Background

In 1989, *Ten Steps to Successful Breastfeeding* (10-step guidelines) was jointly published by the WHO and UNICEF [1]. In 1991, the Baby-Friendly Hospital Initiative was launched [2], under which a hospital that adheres to and promotes the 10-step guidelines as published by the WHO and UNICEF [1], will be certified as a baby-friendly hospitals (BFH). Currently, there are more than 15,000 BHFs worldwide. These hospitals promote breastfeeding, which has unique biological and emotional effects on the health of mothers and babies [3,4].

Japan’s Okayama Medical Center was certified as the first BFH in the developed world in 1991. Since then, the Japan Breastfeeding Association (JBA) has received a mandate from WHO/UNICEF to certify Japanese BFHs [5]. The JBA is the only organization that can certify Japanese BHFs. In spite of these promising beginnings, the number of BFHs in Japan has been slow to increase because the JBA remained a private association until 2010, when it was incorporated as an institution. The JBA has never accepted donations from companies or the dairy industry, and its activity has been limited due to a scarcity of human resources. Within the constraints of its limited resources, the JBA has worked on three pillars of activities: providing BFH certification, hosting training for doctors and midwives, and collecting and providing breastfeeding information. As of December 2010, there were 61 BHF-certified facilities in Japan. At the time of writing, the total number of delivery facilities in Japan is nearly 3,000 [6], which means that BFHs only account for approximately 2% of delivery facilities nationwide.

Japan’s own perinatal care system is unique and elaborate. When a female is diagnosed as pregnant at a hospital, she registers her pregnancy at the municipal government office in the municipality in which she resides. At the time of registration, a maternal and child health (MCH) handbook is given free of charge [7]. This system is in place throughout Japan, where it applies to all of the approximately 1 million annual deliveries. The standard of the MCH handbook is established by the Ministry of Health, Labour and Welfare (MHLW) and every local government arranges their standards to correspond to its contents. The MCH handbook has two functions: to be a record of pregnancy, postparturient status, delivery status of newborns, immunization and child development history up until the age of six years (when children enter elementary school); and as a means of circulating necessary information on child rearing including child development milestones, schedule of immunizations, nutrition and other services offered by local governments. Since Japan’s literacy rate is nearly 100%, the MCH handbook system works well. In almost 99% of cases, the place of delivery is a maternal clinic or hospital [8]. The average length of stay after delivery is 6 days [9]. Newborns are registered with a birth certificate within two weeks of delivery at the local government office. During hospital stay, mothers receive postparturient care including checkups for uterus recovery and breast care, and are instructed how to care for newborns and give breast milk or formula milk as appropriate. Within 4–5 days after birth, babies receive the congenital metabolic disorder screening test, which covers 6 disorders (phenylketonuria, galactocemia, maple syrup urine disease, homocystinuria, congenital hypothyroidism, and congenital adrenal hyperplasia). This test is stipulated by law [10]. After discharge from hospital, public health nurses, who are official staff of the health and welfare sector of the municipal government, make home visits (the newborn visit) - in which they check newborns before the age of one month, hear the complaints of mothers, provide advice and sometimes refer newborns to pediatricians. At one month after delivery, mothers and babies typically consult a doctor for a checkup. This one month check is not subsidized, but mothers routinely visit the clinic or hospital where the infant was delivered. Periodical check-ups for infants are offered free of charge by municipal governments at the ages of 3–4 months, 1.5 years, and 3 years. Regarding child nutrition, public health nurses mainly provide guidance on breastfeeding and weaning to food upon request. Several essential vaccines (DPT, MR, Polio etc.) are also given to children free of charge.

The MHLW’s most recent 10-yearly Child Nutrition Survey (a sampling survey), which was conducted in 2005, listed the nationwide rate of breastfeeding at the age of one month as below 50% [11]. The procedure of the survey is as follows: 3,000 households with infants are randomly sampled by cluster sampling; surveyors visit each of these households and leave self-administered questionnaires, which mothers complete by checking the information recorded in their MCH handbooks. The surveys are collected later. The data collected are then merged and analyzed by the MHLW. The same survey also reported that more than half of the pregnant women surveyed wished to give breast milk to their babies [11]. The discrepancy between the number of mothers who wish to give their babies breast milk and the nationwide goal was reflected in a national campaign for newborn services called “Healthy and Happy Family 21,” in which several discrete goals were set for maternal, newborn, child and adolescent health service activities [12]. The goal for breastfeeding was to increase breastfeeding rate at one month of age from an average of below 50% to 60%. In response to this movement, the JBA has been conducting surveillance on the breastfeeding rate at BFHs since 2007. These hospitals had not been surveyed prior to this time. All BFHs registered in Japan by the JBA are obliged to participate in these surveillance activities. In this article, we summarize the results of this surveillance in order to pave the way to achieving the desired increase from an average of below 50% to 60%, and describe the strength of Japan’s BFHs, which appear to be well supported by their structural and legislative framework. We conclude that a wider implementation of BFH activities to other delivery facilities would be a useful strategy for achieving the desired 60% breastfeeding rate at one month of age.

## Methods

All Japanese BFHs that were recognized as compliant to the Baby Friendly Hospital Initiative standards [13] and which were registered with the JBA were enrolled in this study. A structured questionnaire, which was organized by the JBA, was sent to each registered hospital to collect information about healthy newborns who stayed with their mothers during the delivery period (Table 1). Babies with complications including preterm, low birth weight and cleft palate were excluded from the study as they would most likely have been sent to a hospital with a neonatal intensive care unit or neonatal care unit, which would not be registered as a BFH. The exclusion criteria are listed in Table 1.

**Table 1:** Definition of healthy newborns in the present study

1)	Term baby whose gestational age is between 37 weeks, 0 days and 41 weeks, 6 days.
2)	Birth weight is between 2,500 g and 3,999 g.
3)	Exclusion criteria: Newborns were excluded from the survey if they had the following conditions.
	a) Babies treated separately due to reasons including requirement for incubation or admission to NICU.
	b) Babies given intravenous fluids.
	c) Babies with problems affecting breastfeeding including cleft palate or hypoglycemia.
	d) Babies whose mothers had complications influencing breastfeeding ability including major hemorrhage or the intake of medicines where breastfeeding is prohibited.

The above mentioned questionnaire, the completion of which is mandatory for all BFHs in Japan, is issued annually. Questionnaires are sent to BFHs in January and are returned by April. The contents of questionnaire are as follows: mode of delivery, length of stay and application of labor induction, epidural anesthesia and episiotomy, and the mode of nutrition including supplementation (formula milk or glucose water) during hospital stay, at discharge and at one month of age. The data are collected without unique identifiers (names of mothers or babies, birth dates, addresses, and phone numbers) and the cumulative values for each item are summarized. In the synthesis stage of the questionnaire, we followed the definition of “full” breastfeeding as defined by WHO/UNICEF [14,15], which includes both exclusive breastfeeding and predominant breastfeeding. The number of breastfed children during hospital stay includes the number of fully breastfed children. The number of breastfed children at discharge includes the number of fully breastfed children within 24 hours of discharge. The number of breastfed children at the age of 1 month was determined by interviews carried out in check-ups of one month olds by staff at the respective BFHs, who asked mothers whether or not they were breastfeeding, in consideration of the 24-hour recall recommendation in the indicator guidelines of the WHO [16]. The data were reported in a compiled manner and the breastfeeding rate was calculated based on the collected data. The data were processed and analyzed using Microsoft Excel 2007 (Microsoft, Redmond, WA).

### Ethical considerations

The data were collected only by registered hospitals/clinics and reported to the JBA in a compiled manner. Personal information was not collected (including the names of mothers or babies, birth dates, addresses, and phone numbers). Before launching this study, we consulted the ethical committee of the Nerima Hikarigaoka Hospital and were officially advised that ethical review was not required because the data were collected in an unlinked anonymous manner.

## Results

The data collected covered the years 2007 to 2010. The collection rate for each year was 100%. The numbers of BFHs for each year were 45 (2007), 54 (2008), 59 (2009) and 61 (2010). The number of breastfed newborns ranged from 14,579 (80.2%) in 2007 to 19,209 (73.2%) in 2010. The breastfeeding rate during hospital stay was > 70% (Table 2). The breastfeeding rate at discharge from hospital/clinics reached 90%. The rate at one month was > 75% (Table 2).

**Table 2:** Proportion of breastfed babies

**Year**	**Number of healthy newborns born in BFHs**	**Number of breastfed newborns during hospital stay (%)**	**Number of breastfed newborns at discharge (%)**	**Number of breastfed babies at 1 month (%)**
		**(95% CI)**	**(95% CI)**	**(95% CI)**
**2007**	18,178	14,579 (80.2)	16,803 (92.4)	13,810 (76.0)
		(79.6 to 80.8)	(92.1 to 92.8)	(75.3 to 76.6)
**2008**	23,556	17,668 (75.0)	21,352 (90.6)	19,288 (81.9)
		(75.5 to 75.5)	(90.3 to 91.0)	(81.4 to 82.4)
**2009**	24,032	18,277 (76.0)	21,151 (88.0)	18,893 (78.6)
		(75.6 to 76.6)	(87.6 to 88.4)	(78.1 to 79.3)
**2010**	26,247	19,209 (73.2)	19,210 (73.2)	21,246 (80.9)
		(72.6 to 73.7)	(72.7 to 73.7)	(80.5 to 81.4)

The median length of hospital/clinic stay for normal vaginal delivery was 5 days (minimum/maximum: 5/8) for primipara and 4 days (minimum/maximum: 4/9) for multipara. The median length of stay for cesarean delivery was 10 days (minimum/maximum: 6/15) irrespective of primipara or multipara status.

When breast milk was not sufficiently secreted, glucose water was used more frequently for supplementation than formula milk. The rate of glucose water supplement used was 13.5% in 2007. This increased to 18.8% in 2010. The proportion of formula milk supplementation increased from 8.4% in 2007 to 11.6% in 2008, and increased again to approximately 13% in 2009 and 2010 (Table 3).

**Table 3:** Mode of supplementation

**Mode**	**2007**	**2008**	**2009**	**2010**
	**(n = 18,178)**	**(n = 23,556)**	**(n = 24,032)**	**(n = 26,247)**
**Glucose water, No. (%)**	2,454 (13.5)	3,769 (16.0)	3,845 (16.0)	4,934 (18.8)
**Formula milk, No. (%)**	1,527 (8.4)	2,732 (11.6)	3,124 (13.0)	3,491 (13.3)

**Figure 1:**
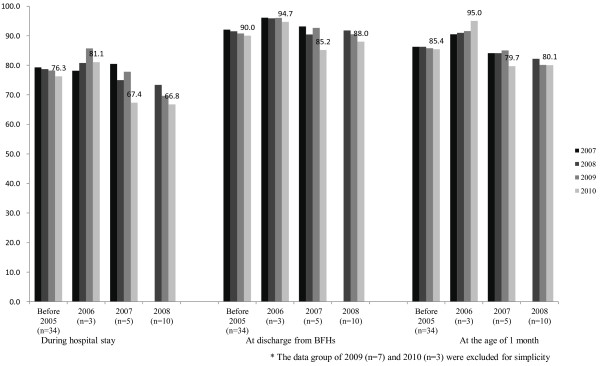
Breast feeding rate trends divided by year of registration.

The breakdown of mode of delivery is shown in Table 4. An increase in the percentage of cesarean section deliveries was observed while labor induction was seen to decrease. When the trends of data were subcategorized by year of registration (34 BFHs registered before 2005, 3 registered in 2006, 5 in 2007, 10 in 2008, 7 in 2009 and 3 in 2010), the breastfeeding rate at discharge was slightly higher for each year than that of the previous year. Interestingly, for each of these years, the breastfeeding rate in the period from discharge to the age of one month, was slightly lower than the previous year (Table 5, Figure 1).

**Table 4:** Summary of mode of delivery

		**2007**		**2008**		**2009**		**2010**
	**No.**	**(%)**	**No.**	**(%)**	**No.**	**(%)**	**No.**	**(%)**
**(1) Total No. of delivery**	23,762		31,036		31,612		35,168	
Single birth	23,088	97.2	30,157	97.2	30,719	97.2	33,622	95.6
Multiple birth*	674	2.8	878	2.8	893	2.8	823	2.8
**(2) Mode of delivery**								
Vaginal delivery	19,146	80.6	24,528	79.0	25,064	79.3	27,454	78.1
Cesarean section	4,505	19.0	6,508	21.0	6,527	20.6	7,670	21.8
Emergency cesarean section**	0	0.0	0	0.0	2,298	7.3	3,387	9.6
No. of vacuum extraction	1,396	5.9	1,902	6.1	1,880	5.9	2,145	6.1
No. of forceps delivery	124	0.5	139	0.4	200	0.6	274	0.8
No. of episiotomy	5,647	23.8	7,700	24.8	7,583	24.0	7,195	20.5
Labor induction	3,453	14.5	5,152	16.6	5,618	17.8	5,385	15.3
Epidural anesthesia***	66	0.3	70	0.2	57	0.2	152	0.4

*Multiple births includes more than twin birth.

**The number of emergency cesarean sections is included in the number of cesarean sections.

***Epidural anesthesia includes painless delivery.

**Table 5:** Breastfeeding rate trends subcategorized by year of registration

	**Year of registration**	**2007**	**2008**	**2009**	**2010**
**Breastfeeding rate during the hospital stay**	Before 2005 (n = 34)	79.3	78.7	78.2	76.3
	In 2006 (n = 3)	78.2	80.8	85.7	81.1
	In 2007 (n = 5)	80.4	75.0	77.8	67.4
	In 2008 (n = 10)		73.4	69.6	66.8
	In 2009 (n = 7)			82.3	82.5
	In 2010 (n = 3)				76.8
**Breastfeeding rate at discharge from hospital**	Before 2005 (n = 34)	92.0	91.5	90.8	90.0
	In 2006 (n = 3)	96.1	95.9	96.1	94.7
	In 2007 (n = 5)	93.1	90.4	92.6	85.2
	In 2008 (n = 10)		91.8	90.5	88.0
	In 2009 (n = 7)			93.4	92.8
	In 2010 (n = 3)				61.8
**Breastfeeding rate at the age of one month**	Before 2005 (n = 34)	86.2	86.2	85.7	85.4
	In 2006 (n = 3)	90.5	91.0	91.6	95.0
	In 2007 (n = 5)	84.1	84.1	85.0	79.7
	In 2008 (n = 10)		82.2	80.1	80.1
	In 2009 (n = 7)			86.3	86.2
	In 2010 (n = 3)				80.8

## Discussion

Our survey describes the current status of breastfeeding in Japan’s BFHs. The breastfeeding rate during hospital stay was more than 70% and reached almost 90% at discharge from hospital/clinics. Even though there was an apparent decrease in the breastfeeding rate at one month, the rate was still higher than the national average. We surmise that Japan’s BFHs nurture better opportunities for breastfeeding.

The main reason for the high breastfeeding rate in Japan’s BFHs can be attributed to the length of stay in the hospital/clinic, which was at least 5 days (median). Within 5 days, almost all mothers experience stage 2 of lactogenesis, in which copious secretion of breast milk begins [17]. The conditions of breast milk production and newborns can be monitored for 24 hours by medical staff. Staff following the 10-step guidelines [1], can thus give appropriate advice to mothers who wish to breastfeed their newborns. Japan’s national median length of hospital stay is 6 days [9], which is almost identical to our result with regard to corresponding to the timing of the inception of lactogenesis. This duration of hospital stay is possible because of a lump-sum allowance for childbirth and nursing, in which the cost of delivery is covered by health insurance. The amount is generally JPY 420,000 (USD 4,200; USD 1 ≈ JPY 100) for each delivery. This allowance is in place for all of the approximately 1 million annual deliveries in Japan [18,19]. Under this allowance, the performance of the congenital metabolic disorder screening test at obstetric hospitals must be carried out before discharge (4–5 days after delivery), thus freeing hospitals/clinics of the burden of babies returning to receive the test.

This structural benefit also applies to supplementation in cases of breast milk shortage during hospital stay. Glucose water is used for supplementation in a higher proportion of cases than formula milk. In the guidelines published by the Academy of Breastfeeding Medicine, the recommended mode of supplementation of milk formula and glucose water is regarded as inappropriate [20]. However, in Japan’s BFHs, glucose water is the major mode of supplementation in cases of shortage of breast milk if the mother is not suffering fatigue or stress. The standard of application of glucose water is stipulated by the JBA Committee of Supplementation [21], and its recommendations do not differ from those of the American Academy of pediatrics [22]. In practice, its application varies among BFHs based on the medical decisions of the doctors/midwives in charge. When doctors or nurses find symptoms (including a more than 10% body weight decrease from birth weight, development of fever without infection, or insufficient breast milk secretion) glucose water supplementation is considered as medical indication and is generally initiated [22]. Glucose water is considered a temporary substitution for breast milk in Japan’s BFHs and mothers with a shortage of breast-milk can use it while they wait for their breast milk supply to become sufficient. This is because medical staff can closely observe the condition of babies and advise mothers until the beginning of breast milk secretion. Here again, the median length of 5 days contributes to a benefit for both mothers and babies. Due to the advice they receive, mothers at BFHs may thus avoid frustration with breastfeeding. According to Watt et al., “It is a matter of opinion to decide the most appropriate length of postpartum in-hospital stay because the length of stay has ranged from 14-day lying-in periods to “drive-through” deliveries with only several hours of postpartum in-hospital care [23]”. Our findings suggest that “drive-through” deliveries are not optimal for the appropriate promotion of breastfeeding. The increase in the number of cases of supplementation with glucose water and formula milk may have a relationship with the increase in the number of cesarean section deliveries. However, it is not possible to confirm this without analyzing individual data, which were not collected in our surveillance. Detailed analysis using individual data and including logistic regression analysis to identify contributing factors is a topic for further research. Regarding the decrease in breastfeeding rate at one month, we speculate that one of the main contributing factors is mothers’ feeling discontent at their level of breast milk secretion [24,25], as well as child rearing stress and the flood of formula milk information. Here again, detailed analysis to identify contributing factors would be an interesting topic for future study.

The existing function of BFHs may be another reason for the high breastfeeding rate. In line with Part 10 of the 10-step guidelines [1], BFHs have an additional role in fostering the establishment of breastfeeding support groups, and to refer mothers to these groups upon discharge from the hospital/clinic. Midwives who spent several days with mothers and developed a trusting relationship can play an important role for referral to support groups [26]. Considering the data trend, BFH registration may not always motivate BFH staff to maintain a high breastfeeding rate because the breastfeeding rates in each group showed a mild decrease after registration. Thus, the promotion of greater adherence to Baby Friendly Hospital Initiative guidelines is something that should be considered. Even though further surveys are needed, we surmise that these activities would support communication between mothers and thus increase the breastfeeding rate.

As seen above, Japan’s perinatal service situation fits well with the BFH services and provides strong support for Japan’s BFH activities. In an article which analyzes policy directions in EU countries, including high performance countries like Sweden and Norway, Cattaneo et al. pointed out that, in order to improve breast-feeding services, it is necessary to use best-evidence-based models, enhance legislative protections and provide more widely-available training [27]. In the case of Japan, it is evident that BFHs in the current framework are the best evidence-based models since supportive legislation already exists. Thus, the wider implementation of Japan’s BFH activities, including the provision of training by the JBA would be a reasonable strategy for increasing the breastfeeding rate. Since 2010, when the JBA became an incorporated body, it has recommended enhancement of breastfeeding policies to the MHLW, which reflect the policies of countries with high breastfeeding rates.

### Limitations

There were a number of limitations to this study. First, our discussion is based on the assumption that all BFH standards are strictly applied by each BFH. The details of services provided at each BFH were not scrutinized in this survey, however, we may assume adherence to these standards because each BFH is subject to regular inspection by the JBA. Investigating the precise level of compliance with BFH standards at each of the facilities will be a further challenge.

Second, we should consider the reliability of national data as a reference. While BFH data is retrieved yearly as an enumeration survey, the most recent national data was acquired in 2005 as a sampling survey and only its estimation was reported. In addition, the national survey, the questionnaire simply asked whether mothers breastfed, provided formula milk or whether they were mixed feeding. This three-way classification (breast feeding, formula milk feeding and mixed feeding) corresponds to the classifications of the MCH handbook. Furthermore, the national data may include babies that are excluded from the BFH data. The application of this kind of data as a reference is not strictly appropriate. We have adopted this data for comparison due to the absence of more appropriate national data, even from research papers. The data were adopted on the basis of the strategies of the “Healthy and Happy Family 21” survey. However, our BFH data were gathered by enumeration surveillance. Since BFHs are considered to be motivated to promote breastfeeding, the results could have a reverse confounding effect. Notwithstanding these limitations, we believe that the data that were utilized are suitable for drawing our conclusions. Our BFH data were sufficiently reliable and while the national data does not allow for the desired level of precision, it is suitable for gaining a reasonable understanding of the breastfeeding situation in Japan.

Third, we did not analyze the reasons for breastfeeding dropout during hospital/clinic stay. The scrutiny of reasons for breastfeeding dropout will be a future challenge. In addition, a detailed analysis of mode of delivery among dropout mothers would be an interesting topic of study.

Fourth, it is impossible to analyze correlations with regard to type of delivery, etc. and type of feeding because the data were reported in a compiled manner. In order to analyze these data, it is necessary to collect a dataset from individual mothers. To accomplish this, we would need to obtain ethical clearance from the respective BFHs. This will be a future challenge for our research.

Finally, as shown in Figure 1, the breastfeeding rate of mothers after they leave the BHF facilities has dropped year-by-year. This may indicate that the high rate of breastfeeding in the BFH is due to selection, rather than the BFH activities. We should consider the reason for this decline in the breastfeeding rate. We speculate that the drop can be attributed to two reasons: the high rate of cesarean section deliveries at BFHs and provider fatigue after BFH certification. According to our data, the percentage of cesarean deliveries increased in 2007 and 2010. Prior et al. pointed out a negative association between cesarean delivery and breastfeeding in their systematic review article [28]. In addition, studies in Sweden and Austria have shown that cesarean section deliveries are linked to greater risk of breastfeeding complications [29,30]. Thus, we speculate that the increase of cesarean delivery is the main contributing factor for the decrease in breastfeeding rate. As Yamada et al. pointed out in their survey of one Japanese BFH, adverse effects of cesarean deliveries may contribute to the increased breastfeeding dropout [31]. In Japan, the proportion of cesarean delivery is gradually increasing [32,33], which may have a negative effect on the national breastfeeding rate. Thus, we should consider special support for mothers who delivered by cesarean section during hospital stay including close counseling, and follow-up care after discharge, including individual home visits for mental support in order to mitigate the collapse of breastfeeding. As for provider fatigue, we speculate that staff at BFHs may experience carelessness after certification. Although a more detailed interview survey would be needed to confirm the extent to which this exists, a training program for staff after certification could be a useful for reducing staff carelessness.

## Conclusions

In our survey of Japanese BHFs, we found that the breastfeeding rate at one month of age was greater than 75%, which surpassed the national average of less than 50%. The median length of hospital stay for delivery at BHFs was 5 days (1 day less than other delivery facilities) is sufficient for copious breast milk secretion to develop in mothers.

The strength of Japan’s BFHs in breastfeeding is that they are supported in both the legislative and structural framework. In the current situation, BFH activities in Japan can play an important role in increasing the breastfeeding rate. Even though BFHs account for only 2% of delivery facilities in Japan, the wider implementation of BFH activities in delivery facilities, with special support to mothers who delivered by cesarean section, would be a useful strategy for achieving the national target of a 60% breastfeeding rate at one month of age.

## Abbreviations

BFH: Baby-friendly hospital; JBA: The Japan Breastfeeding Association; MCH: Maternal and child health; MHLW: Ministry of health labour and welfare.

## Competing interests

The authors declare that they have no competing interests to report.

## Authors’ contributions

TY had a major role in designing the study and data analysis. KT had a major role in data analysis and interpretation of the data, and was the lead writer of this manuscript. YY was co-supervisor of all aspects of study implementation. All authors read and approved the final manuscript.

## Pre-publication history

The pre-publication history for this paper can be accessed here:

http://www.biomedcentral.com/1471-2393/13/207/prepub
